# Hypodermic Injection of Morphia

**Published:** 1879-09

**Authors:** 


### HYPODERMIC INJECTION OF MORPHIA.

Dr. H. H. Kane, of New York city, who has for some time past been collecting statistics on the hypodermic injection of morphia, would consider it a great favor if members of the profession who see this and have had experience with the instrument will answer, by letter to him or otherwise, the following questions:

1. What is your usual dose?
2. Do you use it alone or with atropia?

3. What is the largest amount you have ever administered?

4. Have you had inflammation or abscess at the point of puncture?

5. Have you had any deaths or accidents caused by this instrument?

6. Do you know of any cases of opium habit thus contracted.

Where there has been an autopsy (5) please state the fact and the results obtained therefrom. All communications will be considered strictly confidential, the writer's name being used only when he gives his full consent thereto. Address all letters to Dr. H. H. Kane, 366 Bleecker street, New York.

